# Ayushman Bharat Health Account (ABHA) Integration in Action: Identifying Operational Barriers at a Tertiary Care Center in Eastern India

**DOI:** 10.7759/cureus.103882

**Published:** 2026-02-18

**Authors:** Vikas Dagar, Subhodip Mitra, Ritesh Singh, Ajay Mallick, Rashmi R Guru

**Affiliations:** 1 Pathology, All India Institute of Medical Sciences, Kalyani, IND; 2 Hospital Administration, All India Institute of Medical Sciences, Mangalagiri, IND; 3 Community/Family Medicine, All India Institute of Medical Sciences, Kalyani, IND; 4 Otolaryngology - Head and Neck Surgery, All India Institute of Medical Sciences, Kalyani, IND; 5 Hospital Administration, Postgraduate Institute of Medical Education and Research, Chandigarh, IND

**Keywords:** ayushman bharat health account (abha), digital health awareness, electronic medical records (emr), implementation barriers, institute of national importance, network connectivity, pareto principle, smartphone health applications

## Abstract

Background and aim

The Ayushman Bharat Health Account (ABHA) forms the backbone of India’s digital health mission, enabling the creation of unique, interoperable digital health identities. Despite government mandates, integration at the facility level remains challenging. This study aimed to evaluate the implementation of ABHA creation and linking services at the All India Institute of Medical Sciences (AIIMS), Kalyani, and to identify the principal barriers to successful integration.

Methods

A prospective observational study was conducted from 19 June 2024 to 29 November 2024 in the outpatient department (OPD). Daily data were recorded for the following four predefined hurdles: no smartphone, no signal reception, refusal, and miscellaneous. Descriptive statistics and Pareto analysis were used to identify major contributors to integration failure.

Results

A total of 5,873 (100%) hurdle events were recorded, with the leading barriers being lack of network signal in 2,427 events (41.3%), absence of a smartphone in 1,802 events (30.7%), patient refusal in 1,303 events (22.2%), and miscellaneous factors in 341 events (5.8%). Pareto analysis showed that the top two hurdles, i.e., poor signal reception and lack of a smartphone, accounted for 72.0% of all integration failures (n=5,873). During the study period, the total OPD footfall was 80,497, with 30,786 ABHA tokens generated and 13,085 ABHA linkages completed, yielding an average daily OPD attendance of 1,183.8 patients, with a mean of 427.6 ABHA tokens generated and 181.7 ABHA linkages completed per day.

Conclusion

Poor network connectivity and lack of digital access among patients were the predominant barriers to ABHA integration. Addressing these two core issues could resolve nearly three-fourths of integration failures. Strengthening digital infrastructure and deploying assisted digital registration services are critical to improving ABHA adoption.

## Introduction

Health information systems in developing countries function as “networks of action,” where sustainability depends on continuous local engagement, interactive adaptation, and integration into routine workflows [1]. The Ayushman Bharat Digital Mission (ABDM) aims to create a seamless digital health ecosystem in India through interoperable health records and unique identifiers such as the Ayushman Bharat Health Account (ABHA) number [2]. ABHA enables longitudinal patient records, facilitates continuity of care, and supports telemedicine, insurance claims, and digital health analytics [3].

Agarwal et al. describe digital transformation in healthcare as a shift from paper-based, fragmented systems to integrated digital platforms that improve care quality, efficiency, and data-driven decision-making. The authors emphasize that successful implementation depends not only on technology adoption but also on organizational change, user acceptance, and supportive policy frameworks [4]. In 2013, Scott and Mars reported in the implementation science literature that digital health initiatives in low- and middle-income countries often face barriers related to infrastructure reliability, digital literacy, and trust in data security mechanisms [5]. These factors are particularly relevant in high-volume public hospitals, where operational constraints and patient diversity can directly influence the adoption of digital health.

Ranjan et al. evaluated the efficiency of online ABHA-based outpatient registrations compared to conventional offline methods at a tertiary care hospital. They found that online ABHA registration reduced average registration time from 10.37 min to 4.15 min (offline), representing approximately 60% time savings. The primary bottleneck in offline registration was waiting time (9 min vs. 1 min online). These results demonstrate that ABHA digitization significantly improves registration efficiency and consistency, supporting its broader implementation in healthcare settings [6].

The All India Institute of Medical Sciences (AIIMS), Kalyani, a tertiary care center serving a mixed rural-urban population in Eastern India, initiated a focused ABHA integration initiative across all OPDs. Early observations suggested systemic and patient-related challenges that were slowing adoption. This study was conducted to identify the principal barriers affecting ABHA integration.

## Materials and methods

Study design and setting

This prospective observational study was conducted at the Outpatient Department (OPD) of the All India Institute of Medical Sciences (AIIMS), Kalyani, West Bengal. The study period extended from 19 June 2024 to 29 November 2024. AIIMS, Kalyani is a tertiary-care teaching hospital serving a large patient population from both urban and rural areas, making it an appropriate setting to evaluate real-world implementation challenges of the Ayushman Bharat Health Account (ABHA) system.

Study population

All new OPD patients without a hospital central registration number who approached the ABHA counters during the study period were included. There were no age or gender restrictions. Patients who completed ABHA creation and linkage without difficulty were excluded from hurdle analysis but included in daily OPD footfall, ABHA token generation, and linkage statistics.

ABHA creation and linking workflow

ABHA creation and linking services were provided as per the National Digital Health Mission (NDHM) guidelines using the institute’s Hospital Management Information System (HMIS) integrated with the ABHA platform. Patients were offered ABHA creation or linkage at eight designated registration counters operated by trained staff. Authentication was primarily Aadhaar-based, using a mobile number-linked one-time password (OTP), in accordance with prevailing government protocols.

Data collection and variables

Data were collected daily by trained registration and HMIS staff. For each unsuccessful ABHA creation or linkage, the reason was recorded under four predefined categories identified through a prior questionnaire to ABHA counter staff. First, a lack of smartphone ownership was noted when patients did not possess a smartphone required for OTP-based authentication or app-based ABHA services. Second, a lack of signal reception was documented when poor or absent mobile network connectivity at the point of service prevented successful ABHA creation or linkage. Third, refusal was recorded for patients who were unwilling to create or link ABHA despite counseling, commonly due to lack of interest, privacy concerns, or perceived lack of benefit. Fourth, miscellaneous reasons were documented, including Aadhaar mobile number mismatches, server downtime, technical or software-related errors, incomplete documentation, or patient-related time constraints. In addition, operational metrics were recorded daily, including total OPD footfall, the number of ABHA tokens generated, and the number of ABHA linkages successfully completed.

Statistical analysis

Data were compiled using spreadsheet software and analyzed using descriptive statistics. Frequencies and percentages were calculated for each hurdle category. Mean values were computed for daily OPD footfall, ABHA token generation, and ABHA linkage completion. A Pareto analysis was performed to identify the major contributors to ABHA integration failure, based on the principle that a small number of causes account for the majority of outcomes. Mean values and the Pareto chart were analyzed using Excel 2021 (Redmond, WA: Microsoft Corp.).

Ethical considerations

The study involved analysis of operational and aggregated service-level data without the collection of personal identifiers. No patient-level clinical information was recorded. The study was conducted as part of routine quality improvement and digital health implementation monitoring at AIIMS, Kalyani, in compliance with institutional policies and national digital health guidelines.

## Results

Overall burden of ABHA integration hurdles

During the six-month study period from June to November 2024, a total of 5,873 (100%) ABHA integration-related hurdle events were documented at the OPD registration counters. The overall distribution of these hurdles and the associated operational indicators is summarized in Table 1.

**Table 1:** Summary of ABHA integration hurdles (June-November 2024). ABHA: Ayushman Bharat Health Account

Variables	Total	Mean	Standard deviation
No smartphone	1,802	25.03	11.03
No signal reception	2,427	33.70	20.07
Refusal	1,303	18.10	9.19
Miscellaneous	341	4.74	2.77
Total hurdles	5,873	81.57	33.08
ABHA tokens generated	30,786	427.58	288.55
ABHA linked	13,085	181.74	89.07
OPD footfall	80,497	1183.78	323.94

Among the recorded hurdles, the leading barriers were the lack of network signal in 2,427 events (41.3%), absence of a smartphone in 1,802 events (30.7%), patient refusal in 1,303 events (22.2%), and miscellaneous factors in 341 events (5.8%). The mean number of hurdle events recorded per day was 81.57±33.08.

ABHA operational performance

During the study period, the total OPD footfall was 80,497, with 30,786 ABHA tokens generated and 13,085 ABHA linkages completed, yielding an average daily OPD attendance of 1183.78 patients, with a mean of 427.6 ABHA tokens generated and 181.7 ABHA linkages completed per day, indicating a substantial patient load relative to the number of successful ABHA linkages achieved.

Distribution of individual hurdles

Descriptive analysis revealed that no signal reception had the highest daily mean (33.70±20.07; minimum-maximum: 11-67), followed by no smartphone (25.03±11.03; minimum-maximum: 7-93), refusal (18.10±9.19; minimum-maximum: 3-41), and miscellaneous factors (4.74±2.77; minimum-maximum: 0-11). These findings indicate that infrastructural and access-related issues outweighed behavioral or administrative causes.

Pareto analysis of ABHA integration barriers

**Figure 1:**
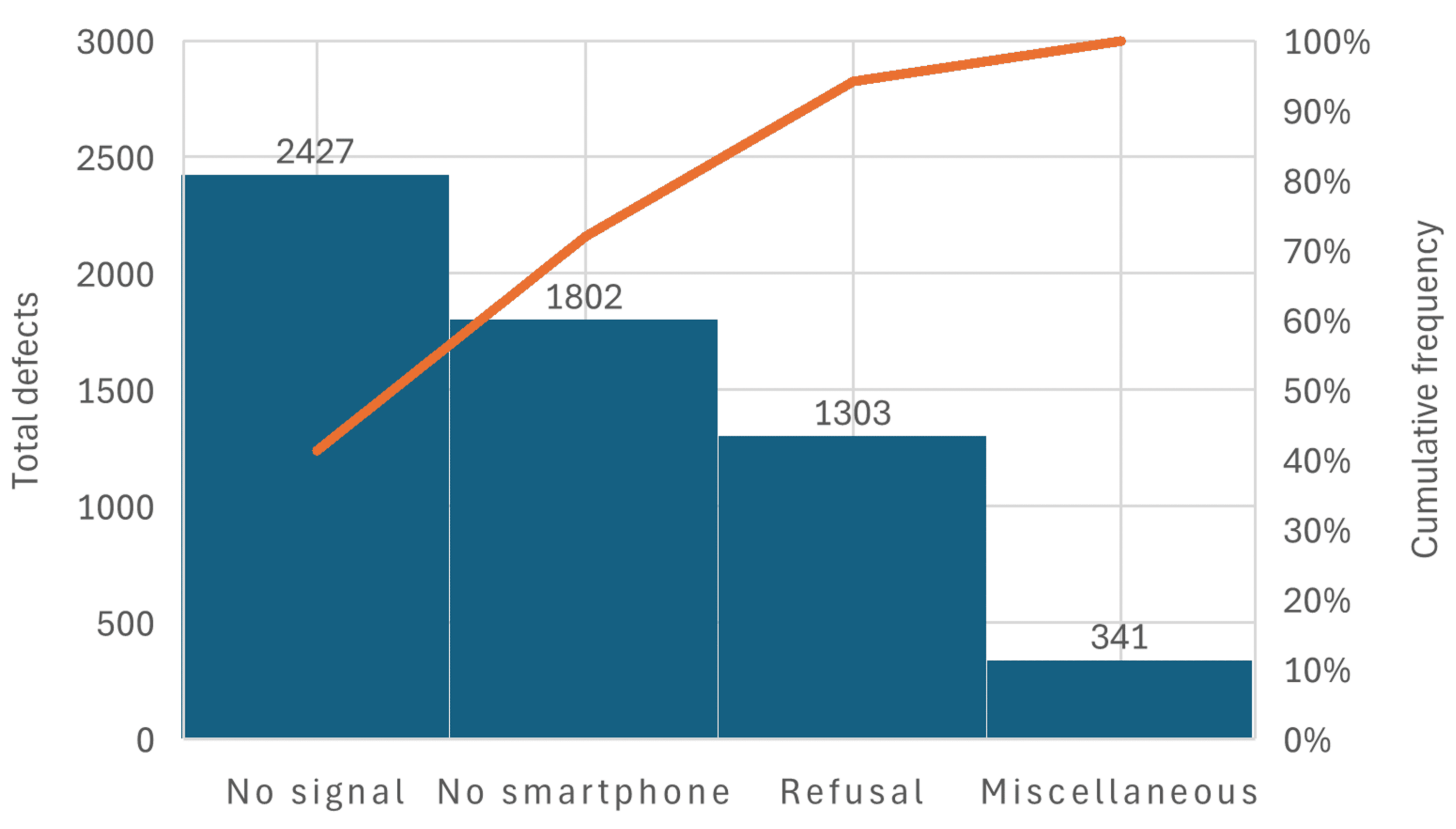
Pareto chart analysis: ABHA non-compliance reasons. ABHA: Ayushman Bharat Health Account

Pareto analysis demonstrated a highly skewed distribution of integration failures (Figure 1). No signal reception contributed 2,427 events (41.3%) of total failures, followed by no smartphone availability in 1,802 events (30.7%), refusal in 1,303 events (22.2%), and miscellaneous factors in 341 events (5.8%).

The cumulative Pareto curve revealed that the top two hurdles, i.e., poor signal reception and lack of a smartphone, accounted for 72.0% of all integration barriers (n=5,873). When refusal was included, the cumulative contribution increased to 94% of all integration barriers (n=5,873), indicating that a small number of high-impact barriers were responsible for the majority of failures.

Interpretation of findings

Network connectivity emerged as the most significant barrier to ABHA integration, reflecting dependence on mobile network availability and local digital infrastructure. The substantial contribution of smartphone unavailability highlights persistent socioeconomic and digital access disparities, which are particularly relevant in semi-rural and resource-constrained settings. Although patient refusal accounted for a smaller proportion of failures, it remains a meaningful barrier, often associated with limited awareness, privacy concerns, and a perceived lack of immediate benefit from ABHA registration.

## Discussion

This study demonstrates that ABHA integration at AIIMS, Kalyani, was substantially affected by both infrastructural and patient-level barriers, with network-related issues emerging as the single most important determinant of integration failure. The predominance of no signal reception (41.3%) as the leading hurdle underscores the critical dependence of digital health systems on reliable telecommunications infrastructure. Similar observations were reported by Gandhi and Soundappan, who evaluated health ID implementation in district hospitals across North India and found that nearly 40% of failed digital registrations were attributable to poor mobile network connectivity [7]. These findings indicate that network-related challenges are systemic rather than institution-specific.

The second most significant barrier identified was lack of smartphone access (30.7%), reflecting persistent digital inequity among healthcare seekers. National surveys such as the National Family Health Survey 5 (NFHS-5) and the National Sample Survey Organisation (NSSO) reports have consistently documented regional and socioeconomic disparities in smartphone ownership, especially among elderly individuals, women, and populations from lower socioeconomic strata [8,9]. A hospital-based study from India by Kumar et al. similarly reported that approximately one-third of patients were unable to complete Aadhaar-linked digital registrations due to the non-availability of personal smart devices [10]. van Dijk highlighted that unequal access to digital devices remains a fundamental limitation to equitable participation in digital systems, particularly among elderly individuals and socioeconomically disadvantaged populations [11]. The concordance of these findings with the present study underscores the structural limitations of smartphone-dependent digital health models.

Although ABHA digitization improves efficiency, several barriers limit its adoption in practice. Limited awareness, poor digital literacy, infrastructure gaps, and data privacy concerns remain major challenges to the implementation of the Ayushman Bharat Digital Mission (ABDM) [12,13]. In our setting, patient refusal (22.2%) was a key cause of ABHA integration failure, consistent with studies that identify fear of Aadhaar misuse, privacy concerns, and perceived lack of benefits as major deterrents. Gandhi and Soundappan emphasized that trust-building and digital literacy interventions are essential to improve acceptance of national digital health identifiers [7]. These findings indicate that technological readiness must be complemented by targeted patient engagement strategies for successful ABDM implementation in public hospitals.

Operational analysis revealed a marked gap between ABHA token generation (mean: 427.6/day) and successful ABHA linkage (mean: 181.7/day). Similar attrition between initiation and completion of digital workflows has been reported by Mishra et al., who observed a 40-60% drop-off rate in ABDM-linked hospital registration processes at tertiary care centres [14]. This attrition reflects inefficiencies introduced by real-time authentication failures, infrastructural constraints, and patient dropouts.

Pareto analysis in the present study demonstrated that the top two hurdles, network issues and lack of smartphones, accounted for nearly 72% of all integration failures, supporting the Pareto principle commonly applied in health systems optimization. Kim et al. demonstrated that lean production offers a promising approach to deliver high-quality, efficient patient care in healthcare, though its implementation poses certain challenges. Given their central role in care delivery, hospitalists are well-positioned to lead the adoption of lean principles to improve quality and efficiency in hospital settings [15]. Similarly, Kruse et al. highlighted that infrastructural reliability and ease of access are foundational prerequisites for sustained digital health adoption, particularly in resource-constrained environments [16]. These findings collectively suggest that focused, context-specific interventions can yield disproportionate benefits.

Based on these insights, localized mitigation strategies are imperative. Provision of hospital Wi-Fi access points, installation of network and Wi-Fi boosters around registration counters, and strengthening backend connectivity can directly address the dominant infrastructural bottleneck. Establishment of ABHA facilitation kiosks and deployment of trained digital counsellors can mitigate device-related exclusion and reduce refusal rates through assisted registration and awareness generation. These interventions are in alignment with ABDM operational guidelines and pilot implementations reported from digitally advanced Indian states [7,12].

Overall, this study provides evidence that barriers to ABHA integration are largely modifiable. Addressing a small number of high-impact challenges could significantly enhance ABHA adoption and linkage rates, thereby strengthening longitudinal digital health records and advancing the objectives of the Ayushman Bharat Digital Mission.

Limitations

This was a single-centre study with a limited observation period; analysis of larger datasets over longer periods is required to draw more robust conclusions. Because the study was conducted in a newly established tertiary care hospital, the findings may differ from those of older, well-established institutions with more mature digital systems. Additionally, urban-rural differences in digital infrastructure and patient digital literacy were not evaluated. Multicentric studies across diverse healthcare settings are needed to better understand these variations and improve the generalizability of results.

## Conclusions

Pareto analysis showed that ABHA integration failures at AIIMS, Kalyani, are driven mainly by a few high-impact barriers, particularly poor network connectivity and lack of smartphone access. Targeted actions, such as installing and strengthening internet infrastructure, deploying network boosters, providing tablets at registration counters, and displaying government-mandated ABHA benefit signboards, can improve adoption, efficiency, and equity in ABHA implementation.

## References

[1] Braa J, Monteiro E, Sahay S (2004). Networks of action: sustainable health information systems across developing countries. MIS Quarterly.

[2] (2019). Government of India, Ministry of Health and Family Welfare, eHealth Section. https://www.mohfw.gov.in/sites/default/files/National_Digital_Health_Blueprint_Report_comments_invited.pdf.

[3] Jha AK, Doolan D, Grandt D, Scott T, Bates DW (2008). The use of health information technology in seven nations. Int J Med Inform.

[4] Agarwal R, Gao G, DesRoches C, Jha AK (2010). Research commentary - the digital transformation of healthcare: current status and the road ahead. Inf Syst Res.

[5] Scott RE, Mars M (2013). Principles and framework for eHealth strategy development. J Med Internet Res.

[6] Ranjan A, Singh G, Singh H, Singh M (2025). Digital transformation of healthcare access: a comparative time series analysis of online versus conventional OPD registrations at a tertiary care hospital. Cureus.

[7] Gandhi AP, Soundappan K (2024). Perception towards electronic health records & uptake of digital health IDs among the urban residents in northern India: a mixed methods study. Indian J Med Res.

[8] (2021). National Family Health Survey (NFHS-5), 2019-21. Mumbai.

[9] National Sample Survey Office (2021). Government of India - Ministry of Statistics and Programme Implementation, Annual Report 2020-2021. https://mospi.gov.in/sites/default/files/publication_reports/Annual_Report_2020-21%20.pdf.

[10] Kumar N, Mehrotra M, Bakshi RK (2023). Factors affecting acceptability of Ayushman Bharat Health Account (ABHA) digital health ID: a multicentre study. Res Rev J Health Prof.

[11] van Dijk JA (2006). Digital divide research, achievements and shortcomings. Poetics.

[12] Kamath R, Banu M, Shet N (2025). Awareness of and challenges in utilizing the Ayushman Bharat Digital Mission for healthcare delivery: qualitative insights from university students in coastal Karnataka in India. Healthcare (Basel).

[13] Arjun MC, Poorvikha S, Kurpad AV, Thomas T (2024). Knowledge, attitude and practice about Ayushman Bharat Digital Mission and digital health among hospital patients. J Family Med Prim Care.

[14] Mishra US, Yadav S, Joe W (2024). The Ayushman Bharat Digital Mission of India: an assessment. Health Syst Reform.

[15] Kim CS, Spahlinger DA, Kin JM, Billi JE (2006). Lean health care: what can hospitals learn from a world-class automaker?. J Hosp Med.

[16] Kruse CS, Karem P, Shifflett K, Vegi L, Ravi K, Brooks M (2018). Evaluating barriers to adopting telemedicine worldwide: a systematic review. J Telemed Telecare.

